# Non-pharmacological Supportive Care Interventions during Immunotherapy for People with Cancer: A Systematic Scoping Review and Future Directions

**DOI:** 10.1007/s11912-025-01714-x

**Published:** 2025-12-06

**Authors:** Morgan J. Farley, Brighid Scanlon, Megan Crichton, Ashley Bigaran, Amy Barnes, Nicolas H. Hart, Patsy M. Yates, Melissa Eastgate, Kim Alexander

**Affiliations:** 1 Centre for Healthcare Transformation, School of Nursing, Queensland University of Technology, Brisbane, Australia; 2 Cancer and Palliative Care Outcomes Centre, School of Nursing, Queensland University of Technology, Brisbane, Australia; 3 Human Performance Research Centre, INSIGHT Research Institute, Faculty of Health, University of Technology Sydney (UTS), Sydney, Australia; 4 Royal Brisbane and Women’s Hospital, Cancer Care Services, Herston, Australia; 5 Wellness and Supportive Care, Olivia Newton-John Cancer & Wellness Centre, Heidelberg, VIC Australia; 6 Department of Surgery, Faculty of Medicine, Science and Dentistry, University of Melbourne, Victoria, Australia; 7 Olivia Newton-John Cancer Research Institute, Heidelberg, VIC Australia; 8 Caring Futures Institute, College of Nursing and Health Sciences, Flinders University, Flinders, SA Australia; 9 Exercise Medicine Research Institute, Edith Cowan University, WA Joondalup, Australia; 10 Institute for Health Research, University of Notre Dame Australia, Fremantle, WA Australia; 11 Faculty of Medicine, University of Queensland, Brisbane, QLD Australia

**Keywords:** Exercise, Dietary interventions, Immune-related adverse events, Gut microbiome, Immune checkpoint inhibitor, Supportive care

## Abstract

**Purpose:**

Immunotherapy has transformed cancer treatment and outcomes. Patients receiving immunotherapy often encounter immune-related and treatment-related adverse events, leading to substantial supportive care needs. Currently, no recommendations exist to guide the use of non-pharmacological supportive care interventions for people with cancer undergoing immunotherapy treatments. This review aims to summarise the available evidence regarding non-pharmacological supportive care strategies to inform future clinical management and research directions.

**Methods:**

Six electronic databases (PubMed, CINAHL, EMBASE, PsycInfo, Web of Science and Scopus) were systematically searched for studies on non-pharmacological supportive care interventions for adults undergoing immunotherapy, published from October 2014 to October 2024.

**Results:**

A total of 5383 studies were screened, with 14 meeting the inclusion criteria. Five were interventional studies and ten were observational. The interventional studies included three physical activity and exercise interventions, two dietary interventions, and one multimodal intervention. Most interventions were found to be feasible, acceptable, and demonstrate preliminary efficacy at improving quality of life, symptom burden, and clinical outcomes. Observational evidence demonstrated associations between physical activity and dietary factors and improved quality of life, reduced symptom burden, and improved clinical outcomes.

**Conclusion:**

Growing observational and preliminary interventional evidence suggests a multimodal supportive care intervention that includes regular symptom monitoring, dietary support and exercise to address the physical and psychosocial needs of cancer patients undergoing immunotherapy may be beneficial. However, further high-quality trials are needed to confirm their efficacy and inform clinical implementation.

**Supplementary Information:**

The online version contains supplementary material available at 10.1007/s11912-025-01714-x.

## Introduction

The emergence of immunotherapy has revolutionised cancer treatment, offering new hope for people with cancer with previously limited therapeutic options [1]. By harnessing the body’s immune system, immunotherapies, such as immune checkpoint inhibitors (ICIs), have shown unprecedented efficacy across multiple cancer types for those who respond to treatment [2]. The success of drugs targeting CTLA-4, PD-1, and PD-L1 has established immunotherapy as a pillar of cancer treatment alongside surgery, radiation, hormone therapy, and chemotherapy [1, 3]. While the use of immunotherapies is rapidly rising, there remains a large proportion of people who fail to respond to treatment, require dose reductions or cease treatment prematurely due to treatment-related toxicities, therefore strategies to improve treatment tolerance and responsiveness to immunotherapies are warranted [1, 4].

Despite the demonstrated clinical success of immunotherapies, their use has been complicated by the emergence of a unique and sometimes unpredictable side effect profile, referred to as immune-related adverse events (irAEs) [4, 5]. Research suggests irAEs of any grade can occur in > 50% of people treated with ICIs and of those 60% discontinued treatment and 76% received steroids [5, 6]. Cancer type, demographics, and lifestyle factors have been shown to influence irAE development e.g., irAE rates are higher in females, those with sarcopenia and low muscle mass, body mass index > 25 kg/m², lower activity of daily living score, higher pain score, and those on a combination therapy [7, 8]. While immunotherapies are effective at treating some cancers, they subsequently produce a sequalae of physical and psychological disturbances that not only impact quality of life (QoL) but may also lead to dose-reduction, treatment interruption or discontinuation, potentially compromising therapeutic outcomes [5, 9].

Current management approaches for irAEs are informed by the Common Terminology Criteria for Adverse Events (CTCAE) grading scale and primarily rely on systemic corticosteroids, treatment breaks, or discontinuation which can result in significant side effects or interfere with anti-tumour immune responses [10]. Given responsiveness to ICIs relies on an intact and robust immune response, the frequency of use and immunosuppressive nature of corticosteroids has raised questions about whether they may interfere with their therapeutic effect [11]. Moreover, while treatment discontinuation and the administration of corticosteroids may successfully treat and mitigate the risks of irAEs, these management strategies present significant challenges for patients, including the additional short- and long-term side effects of corticosteroid use that can further compromise QoL and treatment efficacy [11].

Supportive care interventions are defined by the Multinational Association of Supportive Care in Cancer (MASCC) as those which prevent and manage the adverse effects of cancer and its treatment [12]. This includes management of physical and psychosocial problems across the survivorship continuum (i.e., from diagnosis to the end of life) [13]. Physical activity (PA) and exercise, diet, and psychosocial support have been demonstrated as effective management strategies in other systemic therapies (such as chemotherapy) to reduce treatment-related toxicities and improve treatment completion rates, QoL, and potentially reduce healthcare utilisation rates [14, 15]. However, findings from studies of patients receiving other therapies do not necessarily translate to immunotherapy due to the distinct pathophysiology of irAEs, which arise from immune system hyperactivation [1]. Regardless, a recent large-scale cross-sectional multinational study mapping the unmet needs of cancer survivors identified 26% of people surveyed had low QoL and the most frequent reported symptoms were fatigue (67%), loss of strength (62%), pain (62%), sleep disturbance (60%), and weight changes (58%) [16]. Each of these effects can be addressed effectively through supportive care strategies.

There is growing interest in supportive care strategies for people with cancer undergoing immunotherapy given their unique side effect profile and emergence of unmet needs. Despite the recognised benefits of supportive care interventions for people receiving other cancer treatments, it is unclear whether supportive care interventions are feasible, acceptable, and effective for people with cancer undergoing immunotherapy [9]. Therefore, this scoping review aims to systematically examine current evidence regarding non-pharmacological supportive care interventions for people undergoing immunotherapy and to identify future directions for research and clinical care.

## Methods

### Literature Review Process

A systematic scoping review was performed in compliance with the framework developed by Arksey and O’Malley [17] and guidelines provided by Joanna Briggs Institute (JBI) Reviewers Manual [18]. In accordance with the JBI Reviewers Manual, a detailed protocol was created but not registered for this review [18]. The review is reported according to the Preferred Reporting Items for Systematic Reviews and Meta-analyses Extension for Scoping Reviews (PRISMA-ScR) Checklist (Supplementary Material 1) [19]. Supportive care interventions were defined as per MASCC’s definition [12, 20]. Probiotic-based interventions were also excluded as this has been reviewed elsewhere [21].

#### Review Objectives and Aims

The primary aim of this review was to systematically identify non-pharmacological interventions reported within interventional studies to manage toxicities and improve outcomes for people with cancer receiving ICIs. The secondary aim was to summarise the observational evidence base to inform future directions for non-pharmacological supportive care interventions.

#### Inclusion Criteria

To be included in this review, articles were required to:

1. Explore the effect a non-pharmacological supportive care intervention for the management of immunotherapy-related toxicities, symptom burden, QoL, or treatment outcomes for individuals undergoing immunotherapy for cancer.
2. Have > 50% of the population undergoing immunotherapy for cancer.
3. Primary research articles.
4. Published between 2014 and 2024.
5. Published in English language.
6. All study designs including randomised and non-randomised controlled trials, and single arm studies.

#### Exclusion Criteria

Studies were excluded if they:

1. Reported exclusively on pharmacological interventions (i.e., prescribed and non-prescribed medications, not including dietary and nutrient supplements).
2. Exclusively qualitative methods.
3. In conditions other than cancer.
4. Reported on those aged under 18 years.
5. Conference abstracts or protocol papers.

#### Information Sources and Search Strategy

A systematic search was conducted in October 2024 with the support of an expert Librarian. Searches of the Bibliographic databases PubMed, CINAHL, EMBASE, PsycInfo, Web of Science and Scopus were performed to identify relevant papers published in the previous 10 years. This time frame was selected to align with the recent emergence of immunotherapy. Examples of MeSH search terms included “Immunotherapy”, “Supportive Care”, “Non-Pharmacological Interventions”, “Immune Checkpoint Inhibitors”, “Symptom Burden”, and “Quality of Life” “Physical Activity”, “Diet”, “Nutrition”, “psychology”. Grey literature, such as government reports and clinical guideline databases, were not included [22]. The final search strategy can be seen in Supplemental Material 2.

#### Data Screening, Extraction and Analysis

Included articles were exported into Endnote 21 with duplicates removed [23]. The remaining articles were screened via Covidence software [24]. Two reviewers (MF and BS) independently screened all title, abstracts, and full text articles against inclusion criteria. Disagreements were resolved via a third reviewer (KA).

A data extraction tool was developed by two authors (MF and BS) based on the primary review question and JBI Guidelines [18], and refined by piloting on five studies. The data extraction tool obtained the following information: author, title, year, location, patient population (age, gender, cancer type), immunotherapy treatment, setting (e.g., telehealth, community based, home based), study information (e.g., duration, sample size), intervention details, outcome (feasibility and efficacy) measures, and key findings. Studies were organised by intervention modality (e.g., diet, exercise, or mixed). Quantitative and qualitative synthesis of the extracted data was performed. Consistent with the purpose of scoping reviews, no quality or bias assessments were conducted for individual studies [17].

## Results

**Fig. 1:**
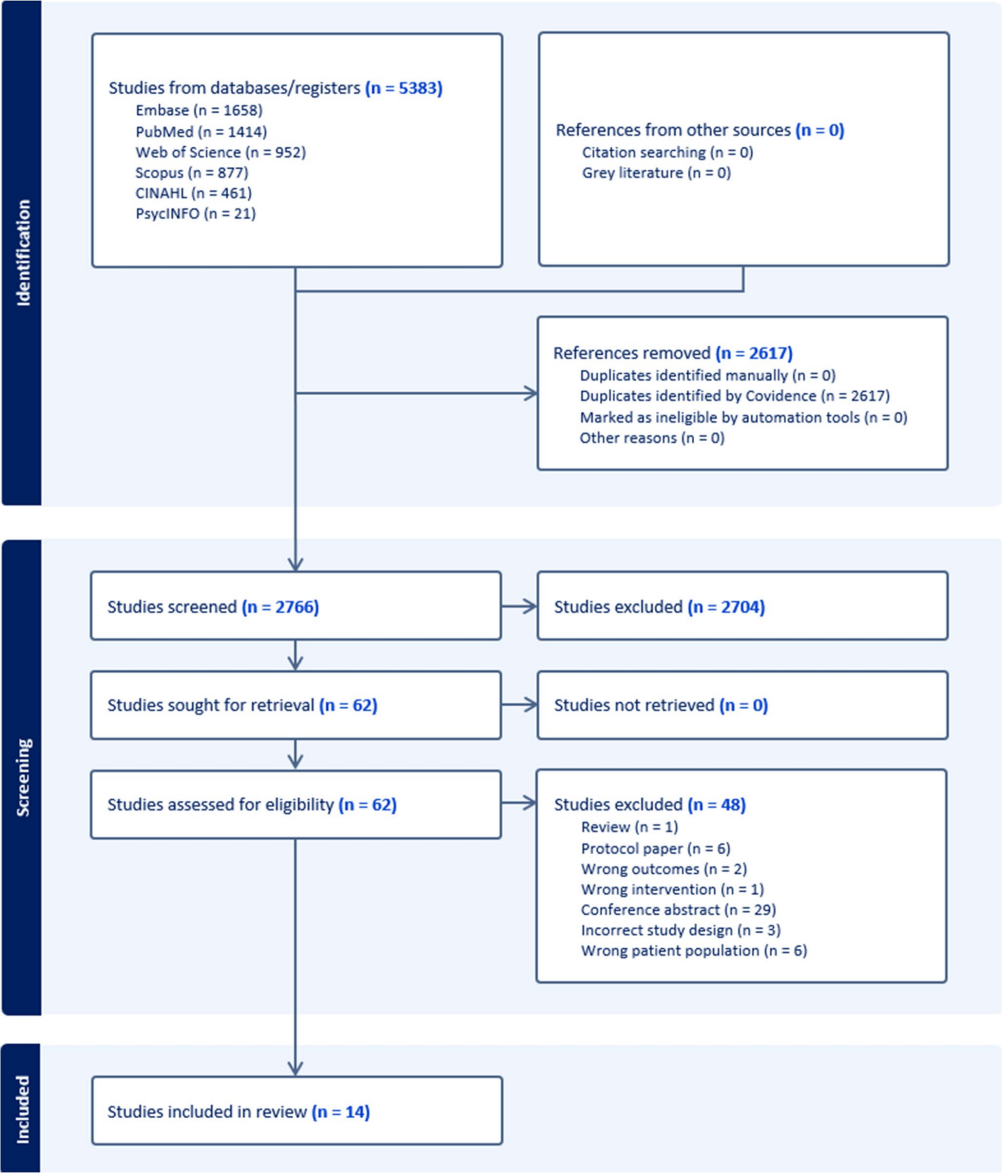
PRISMA flow-diagram including screening and reasons for exclusion during second round of title and abstract screening

The systematic search strategy and screening process, presented in Fig. 1, led to a total of fourteen papers included in this review. Of those included, five were interventional studies and ten were observational. Of the interventional studies, three were PA/exercise feasibility trials [25–27], one examined a multimodal supportive care intervention [28] and one examined Vitamin D supplementation [29]. Tables 1 and 2 report on the characteristics and results of interventional studies. Of the observational studies, four reported on PA/exercise [30–33] and five examined dietary patterns [34–38]. Observational study characteristics and outcomes are reported in Table 3.

**Table 1:** Characteristics of included interventional studies

Authors	Title	Year	Location	Patient population	Immunotherapy treatment	Primary aims	Study design	Sample size	Health setting	Intervention	Control/ comparison	
***Exercise and Physical Activity ***												
Charles et al. [25]	Delivering adapted physical activity by videoconference to patients with fatigue under immune checkpoint inhibitors: Lessons learned from the PACTIMe-FEAS feasibility study	2021	France	Cancer patients on ICIs and experiencing fatigue	Nivolumab or Pembrolizumab	*Pa*: Evaluate the feasibility and acceptability of a videoconference-based program promoting PA. *Sa*: Assess its efficacy in reducing fatigue in cancer patients under ICIs.	Feasibility trial	16	Community level	6-month online PA program delivered Prescription: F: ≥150 min p.w I: moderate Ti: 45–60 min Ty: aerobic, resistance, stretching P: 15-min p.w	None	
Crosby et al. [27]	Feasibility of supervised telehealth exercise for patients with advanced melanoma receiving checkpoint inhibitor therapy	2023	Australia	Advanced melanoma patients on ICIs	PD-1, CTLA-4, or PD- 1/CTLA- 4 combination	Determine the feasibility, safety and explore the preliminary efficacy of a telehealth supervised exercise program in patients with melanoma receiving ICIs.	Non-randomised feasibility pilot trial	11	Community level	8-week combined exercise program delivered via telehealth. Prescription: F: 3p.w I: moderate-high Ti: ≤60 min Ty: AE, RT, BT P: ↑ weight if more reps can be completed	None	
Caballero-Borrego et al. [26]	Walking one hour per day and the derived neutrophil-to-lymphocyte ratio are associated with outcome in palliative second-line immunotherapy for patients with recurrent and/or metastatic squamous cell carcinoma of head and neck	2024	Spain	Recurrent Head and Neck cancer patients on second line immunotherapy	PD-1/PD-L1 inhibitors (nivolumab, durvalumab, and avelumab)	To determine whether routinely walking activity and the derived neutrophil-to-lymphocyte ratio are associated with outcomes in patients with recurrent and/or metastatic squamous cell carcinoma of head and neck.	Non-randomised study	64	Tertiary level	Completion of one hour of uninterrupted walking per day.	Those who completed one hour of uninterrupted physical activity per day vs. those who performed no activity	
***Diet***												
Galus et al. [29]	Vitamin D supplementation increases objective response rate and prolongs progression-free time in patients with advanced melanoma undergoing anti–PD-1 therapy	2023	Poland	Advanced Melanoma patients undergoing anti-PD-1 therapy	pembrolizumab or nivolumab	To compare the efficacy of anti–PD-1 therapy in patients with locally advanced, inoperable, or metastatic melanoma in relation to serum vitamin D concentrations.	Non-randomised study	200	Tertiary level	Vitamin D Supplementation as a prophylactic dose (2000 IU) or therapeutic dose (4000–6000 IU)	No supplementation	
***Multimodal***												
Lacey et al. [28]	A supportive care intervention for people with metastatic melanoma treated with immunotherapy: a pilot study assessing feasibility, perceived benefit, and acceptability	2018	Australia	Metastatic Melanoma patients on immunotherapy	Pembrolizumab	To assess the feasibility of a multimodal supportive care program to MM patients being treated with pembrolizumab.	Pre-post-test feasibility cohort study	28	Tertiary level	A tailored multimodal 8-week supportive care program: physician consult, exercise, dietary advice, complementary therapies, psychology consultation.	Multimodal intervention vs. no intervention (standard care)	

*PA* Physical activity, *p.w* per week, *F* frequency, *I* intensity, *Ti* time, *Ty* type, *P* progression, *ICIs* immune-checkpoint inhibitor therapy, *AE* aerobic training, *RT* resistance training, *BT* balance training, *Pa* primary aim, *Sa* secondary aim

**Table 2:** Intervention outcomes

Reference	Outcome measures	Feasibility	Acceptability	Efficacy/Clinical outcomes	Recommendations from authors
***Exercise and Physical Activity***					
Charles et al. [25]	• Feasibility (Recruitment Process, Attrition Rate). • Acceptability (Adherence, Satisfaction) • Efficacy (Post-Immediate Fatigue Reduction, Short-term fatigue reduction, physical condition, PA and sedentariness, QoL, PreTrial Exercise Behaviour, Anxiety and Depression, Sleep Disorders)	Recruitment Rate: 44% Attrition Rate: 43.7% Adverse Events: No Severe AE’s	Adherence: 78% (supervised component) Satisfaction: High	Fatigue 2.1-point mean ↓ fatigue ↑ PA time, Intrinsic motivation, endurance, balance and lower limb strength, perceived physical and mental health.	• Future large-scale trials require optimisation of the recruitment process • Qualitative research to provide information on the conditions for adherence to the program • Use of videoconference should be more extensively considered to support regular PA as close to patients’ living conditions
Crosby et al. [27]	• Feasibility (Recruitment Rate, Completion Rate, Safety) • Acceptability (Attendance, Compliance, Tolerance) • Efficacy (Physical Assessments; 2-minute step test, chair stand test, 30-s push up test, ESSA Static Balance Test)	Recruitment Rate: 48% Completion Rate: 91% Safety: No Severe AE’s	Attendance: 88% Compliance: 82.1% (median exercise), 84.9% (resistance and aerobic) Tolerance: 88%	Physical Assessments: ↑ physical functioning; ↑ cardiovascular capacity, ↑upper body strength/endurance, ↑ static balance, ↑ functional performance, QoL maintained post intervention	• Findings to inform future randomised controlled trials with larger sample sizes, a usual care control group, a longer exercise intervention and objective measures of body composition
Caballero-Borrego et al. [26]	• Blood count (derived neutrophil-to-lymphocyte ratio) • Clinical response (OS, PFS, and radiological assessments)			Blood cell count: ↓dLNR (< 3.5) independently associated with ↑ OS (*P* = 0.01. Physical activity independently associated with ↑ PFS (P = < 0.00) and ↑ OS (P = < 0.00)	• Studies with greater control of variables and larger sample sizes are needed to further explore the relationship between exercise and survival • Potential to use dLNR as a tool when selecting immunotherapy patients
***Diet***					
Galus et al. [29]	• Serum Vitamin D levels (ng/mL) • Clinical outcomes (complete response, objective response rate, progression of disease, partial response, stabilisation of disease)			↑ RR, ↑ PFS and ↑ OS in those with normal Vitamin D levels as baseline or via supplementation	• Maintaining normal Vitamin D levels during anti-PD‐1 immunotherapy should be standard practice for advanced melanoma patients
***Multimodal***					
Lacey et al. [28]	• Feasibility (Adherence, Safety) • Acceptability (Completion) • Efficacy (Use of complimentary therapies, Symptom-Burden, QoL,	Overall exercise adherence: 85%, irAE’s were low	Completion of program: 50%	↑ Uptake of complimentary therapies (85%)	• Holistic supportive care intervention should be explored in populations with higher symptom burdens

AE’s: adverse events, PA: physical activity, dLNR: derived neutrophil-to-lymphocyte ratio, OS: overall survival, PFS: progression-free survival, RR: response rate, irAEs: immunotherapy-related adverse events, QoL: quality of life, ESSA: Exercise Sports Science Australia

**Table 3:** Observational evidence for physical activity, dietary factors, and emotional wellbeing and clinical outcomes

Author	Title	Year	Location	Study design	Sample size	Population (Cancer type, treatment type)	Exposure	Outcome (Clinical measurement)	Key findings
***Exercise and Physical Activity***									
Min et al. [30]	Effect of physical activity on patients of NSCLC	2024	China	Cohort study	121	NSCLC Immunotherapy	Physical activity (Light, moderate, vigorous) and sedentary time	irAEs	PA ICI efficacy and irAES (*p* > 0.05) Between group differences in baseline haemoglobin levels (*p* = 0.037) and lymphocyte levels (*p* = 0.047). Between group differences in post-treatment albumin levels (*p* = 0.012) and lymphocyte counts (*p* = 0.035). Negative correlation pre-treatment sedentary duration and ICI efficacy (*p* = 0.027).
Verheijden et al. [31]	Physical activity and checkpoint inhibition: association with toxicity and survival	2023	Netherlands	Cohort study	251	Melanoma, NSCLC, RCC, Other ICI’s (anti-PD- L1, dual-ICI, or ICI + chemo or targeted therapy)	Physical activity (Light (< 3.0 METs), moderate (3.0 to 5.9 METs) or vigorous (≥ 6.0 METs))	Severe irAEs Overall survival	Moderate & vigorous PA associated with ↓ severe irAE compared to light PA (*p* = 0.012) Moderate & vigorous PA associated with ↑ survival (*p* = 0.036)
Liu et al. [32]	Physical activity improves outcomes of combined lenvatinib plus anti-PD-1 therapy in unresectable hepatocellular carcinoma: a retrospective study and mouse model	2022	China	Cohort study	59	Liver cancer Lenvatinib plus anti-PD-1 therapy	Physical activity (active vs. sedentary)	Overall survival Objective Response Rate Progression-free survival	Active group associated with ↑OS (*p* < 0.05) and PFS (*p* < 0.001) and ORR (*p* < 0.01) compared to sedentary Regular PA was independently associated with ↑ OS, PFS and ORR.
Hyatt et al. [33]	Exercise Behaviours and Fatigue in Patients Receiving Immunotherapy for Advanced Melanoma: A Cross-Sectional Survey via Social Media	2019	Australia	Cross-sectional survey	55	Melanoma Immunotherapy (single agent, combination)	Exercise behaviours, number of exercise activities weekly (1–6)	Side effects (fatigue, dermatological, gastrointestinal, endocrine, respiratory, musculoskeletal, neurological, other)	56% described exercising while receiving IO Walking most common activity (77%). Most common benefit of exercise was fatigue reduction 69% mentioned fatigue as a barrier to exercise and ADLs
***Dietary Factors***									
Bolte et al. [34]	Association of a mediterranean diet with outcomes for patients treated with immune checkpoint blockade for advanced melanoma	2023	Netherlands and UK	Cohort study	91	Melanoma Immune Checkpoint Inhibitors	Mediterranean Diet (food frequency questionnaires)	Overall response rate Progression-free survival at 12 months Immune related adverse events (≥ Grade 2)	Positive association between Mediterranean dietary patterns (high in whole grains, fish, nuts, fruit, and vegetables) and ORR (*p* = 0.02) and PFS (*P* = 0.01)
Bersanelli et al. [35]	Systematic vitamin D supplementation is associated with improved outcomes and reduced thyroid irAEs in patients with cancer treated with ICIs	2023	Italy	Cohort study	164	All cancers Immune Checkpoint Inhibitors	Serum Vitamin D	Survival Time to treatment failure Disease Control Rate Toxicity outcomes	Vitamin D supplementation was associated with ↑ OS (*p* = 0.013), TTF (*p* = 0.017), DCR, (*p* = 0.016) compared to a retrospective control
Golcic et al. [36]	Analysis of the Gut Microbiome and Dietary Habits in Metastatic Melanoma Patients with a Complete and Sustained Response to Immunotherapy	2023	Croatia	Cross-sectional study	15	Melanoma Immunotherapy	Gut microbiome Dietary habits	Response rate	Complete response > 9 months (late responders) was associated with ↑ beta-diversity (*p* = 0.02), ↑ abundance of *Coprococcus comes* (*p* = 0.010), *Bifidobacterium pseudocatenulatum* (*p* = 0.024), and ↓ abundance of *Prevotellaceae* (*p* = 0.04) compared to early responders. Late responders had a significantly ↓ intake of proteins & sweets and a ↑ intake of flavones (*p* < 0.05).
Simpson et al. [37]	Diet-driven microbial ecology underpins associations between cancer immunotherapy outcomes and the gut microbiome	2022	Australia, Netherlands, United States	Cohort study	218	Melanoma Immunotherapy	Microbiome metrics (faecal microbiota signatures, dietary patterns)	Response rate Immune related adverse events	↑RR in Ruminococcaceae dominated microbiomes (*p* < 0.0001) compared to Bacteroidaceae dominated microbiomes (*p* > 0.05) ↓response was associated with dietary ↓fibre (*p* = 0.0309) & omega 3 (*p* = 0.0139) and ↑C-reactive protein concentrations
Spencer et al. [38]	Dietary fibre and probiotics influence the gut microbiome and melanoma immunotherapy response	2021	United States	Cohort study	128	Melanoma Immunotherapy	Dietary fibre Probiotic supplement use	Progression-free survival	↑dietary fibre was associated with ↑ PFS (*p* = 0.04) ↑fibre intake and no probiotic use was significantly associated with RR (*p* = 0.03) After adjustment for every 5-g ↑ fibre intake 30% ↓ risk of progression or death

*NSCLC* non-small cell lung cancer, *PA* physical activity, *irAEs* immune-related adverse events, *ICIs* immune checkpoint inhibitors, *RCC* renal cell carcinoma *IL* interleukin, *TNF-α* tumour necrosis factor – alpha, *OS* overall survival, *PFS* progression free survival, *ORR* objective response rate, *METs* metabolic equivalent of task, *ADLs* activities of daily living, *DCR* disease control rate, *QoL* quality of life, *HRQoL* Health-related quality of life, *RR* response rate, *NLR* neutrophil to lymphocyte ratio, *TTF* time to treatment failure, *OES* oesophageal, *CRC* colorectal cancer, *ORR* objective response rate, *DCR* disease control rate

### Interventional evidence and outcomes

#### Exercise Interventions

Of the five interventions identified, three investigated the safety, feasibility, and efficacy of exercise. All studies were delivered online, two prescribed a combined aerobic and resistance training intervention overseen by an Accredited Exercise Physiologist (AEP) [25, 27] and the third prescribed self-directed uninterrupted walking (1 h) [26]. Participants in the intervention by Charles et al. [25] underwent weekly 45–60-minute video-delivered telehealth exercise sessions, which included a combination of aerobic, resistance, and mobility exercises and were provided guidance to achieve 150-minutes of self-directed moderate-vigorous exercise per week for 6-months. Adherence was self-monitored via a logbook and two check-in phone calls with an AEP over six months. The second study utilised a supervised 8-week thrice weekly combined aerobic and resistance exercise training intervention [27]. Participants were prescribed 2–3 sets of 8–12 repetitions for each exercise for six exercises and the aerobic component included ~ 20 min of moderate-to-high intensity exercise (5 sets of 1-min intervals with 30 s rest), adherence was assessed by the AEP [27]. Caballero-Borrego et al. prescribed an entirely self-directed aerobic intervention inclusive of daily uninterrupted walking for 1-hour [26].

### Safety and Feasibility

All PA/exercise interventions reported on the delivery of an online program to be safe, with no severe AEs associated with the intervention. The study by Charles et al. reported challenges with recruitment, with 11.7% of patients contacted being eligible for the trial, of which 71% consented to the study [25]. Reasons for exclusion include ECOG status, age, contraindications to exercise, and lack of information technology support. There was a withdrawal rate of 33% with the most common reason being disease progression. Despite this challenge, those who completed the intervention reported high levels of adherence (78%) to the supervised sessions. Participants also had high levels of satisfaction, expressing they would recommend the program to others and enjoyed the supervised group format [25]. In contrast, Crosby et al. demonstrated a supervised multimodal exercise intervention was safe and feasible with a recruitment rate of 48%, completion rate of 91%, program attendance of 88%, and tolerance of 88% [27]. Notably their intervention was fully supervised and of the missed sessions, 63% were due to treatment-related side effects. Of those who were recruited for the trial by Caballero-Borrego et al. only 44% (*n* = 28) completed ≥ 1 h/day of prescribed uninterrupted walking thus included in the walking group [26]. The primary reason for not participating was treatment-related side effects. No adverse events were reported.

### Efficacy

Both interventions by Charles et al., [25] and Crosby et al. [27] demonstrated significant improvements in patient-reported physical, psychosocial, and fatigue outcomes. Charles et al. observed improvements in fatigue (2.1-point mean decrease in fatigue (*p* = 0.0161; IC: [− 3.61; −0.55]), moderate PA time, endurance, balance, lower limb strength, and overall perceived physical and mental health. Similarly, Crosby et al., observed significant improvements in cardiovascular capacity (*p* < 0.001), upper body strength and endurance (*p* = 0.01), functional performance (*p* = 0.001) and static balance (*p* < 0.001) [27]. Caballero-Borrego et al. demonstrated significant improvements in progression free survival (PFS) (9 months vs. 2 months, *p* = 0.001) and overall survival at 25 months (*p* = 0.001) for those in the walking group [26].

### Dietary strategies

One study examined the role of dietary Vitamin D supplementation on objective response rate and progression-free survival in metastatic melanoma patients [29]. Serum Vitamin D was measured in 200 participants during first-line immunotherapy, an historical control was used. Those with normal baseline concentrations of Vitamin D received a prophylactic dose and those with reduced levels received a therapeutic dose. The authors did not report any safety concerns. There was an increase (36% vs. 56%, *p* = 0.01) in response rate (RECIST 1.1), overall- (31.3 vs. 27 months, *p* = 0.39), and progression free-survival (5.75 vs. 11.25 months, *p* = 0.03) for low vs. high vitamin D at baseline. There were no significant differences in rates of irAEs of any grade.

### Multimodal interventions

One study examined a multimodal supportive care intervention for people with metastatic melanoma undergoing immunotherapy [28]. The intervention comprised of a comprehensive medical assessment by a supportive care physician, an AEP, and Accredited Practicing Dietician. This is the only intervention identified that comprehensively assessed and managed symptoms using a tailored supportive care program including exercise, dietary advice, non-invasive complementary therapies, and psychology. Lacey at al., demonstrated the Living well program to be feasible in this population. All participants were prescribed 16 tailored exercise sessions (aerobic and/or resistance training, group classes, home programs) achieving an 85% adherence rate, 85% of participants accessed between two and 18 integrative oncology sessions with the most common being massage, reflexology, mindfulness, and meditation. Two people (15%) accessed psychological services. Symptoms most troubling at baseline were fatigue (*n* = 6), sleep (*n* = 6), general aches and pains (*n* = 5), and memory (*n* = 4). The frequency of some treatment-related side effects reduced post intervention (fatigue: 50%, sleep: 85%, general aches and pains: 100%). The authors did not perform statistical analyses on efficacy measures (strength, body composition, steps, cardiorespiratory fitness, daily hours of exercise, and symptoms).

### Observational evidence and outcomes

#### Exercise

Three cohort studies and one cross-sectional survey were identified that examined the relationships between ICIs outcomes, PA, and exercise training [30–32]. All studies assessed PA or exercise (sedentary, light, moderate, or vigorous activity) during ICI treatment. Three studies examined associations between exercise and clinical outcomes, and one examined the association between exercise behaviours and fatigue during treatment [33]. Moderate-vigorous PA was associated with a reduction in irAE’s [30] and increased overall- and progression free-survival [31].

### Diet

Five observational studies examined dietary intake and patterns. This included assessment of the Mediterranean diet [34], vitamin D supplementation [35], and dietary patterns promoting microbiota diversity, including dietary fibre and omega-3 polyunsaturated fatty acid intake [36–38]. Adherence to the Mediterranean diet was positively associated with overall response rate (*p* = 0.02) and progression-free survival (*p* = 0.01) [34]. Similarly, Vitamin D supplementation was associated with improved overall survival, time to treatment failure, and disease control rate (all: *p* = 0.01) [35]. Of dietary exposures, dietary patterns promoting microbiota diversity were most frequently reported, particularly dietary fibre intake. High dietary fibre diets and probiotic use were associated with longer progression free survival (*p* = 0.04) and greater response rate (*p* = 0.03) [38], whereas reduced dietary fibre was associated with reduced treatment response (*p* = 0.03), and increased inflammation (*p* = 0.01) [38]. Furthermore, every 5 g increase in daily dietary fibre intake corresponded with a 30% increase in progression-free survival (*p* = 0.04) [38]. Simpson et al. [37] also found lower dietary fibre intake and omega-3 consumption to be associated with poorer responses to treatment. Similarly, Golcic et al. found significant positive correlations between Mediterranean diet concepts and beneficial microbiota (e.g., *Bifidobacterium pseudocatenulatum* species) as well as increased gut microbiota diversity and a complete response of > 9 months (*p* = 0.02) [36].

## Discussion

The field of immunotherapy is evolving, offering potential benefits for patients with traditionally limited treatment options. However, immunotherapy is associated with a sequalae of adverse side effects due to over-activation of the immune system [1]. While non-pharmacological supportive care interventions have shown efficacy at improving QoL, symptom burden, and clinical outcomes for those undergoing traditional systemic anti-cancer therapies [14], there remain significant unmet supportive care needs (e.g., psychological and physical) for those undergoing immunotherapies [39]. As such, this review aimed to identify the current non-pharmacological supportive care interventions available to manage toxicities and improve outcomes during immunotherapy. The secondary aims were to summarise the observational evidence to collectively inform future directions and the design of a supportive care intervention for people undergoing immunotherapy.

This review identified five supportive care interventions: three focused on PA/exercise, one dietary, and one multimodal intervention (exercise, diet, and psychological support) as well as a suite of complementary therapies. Notably, only one intervention included comprehensive symptom assessment [28], the remainder reported on feasibility and clinical outcomes. There is a clear lack of randomised controlled trials (RCT) currently available within the literature, reflecting the emerging nature of supportive care research in immunotherapy.

Although these interventions show promise at improving clinical outcomes such as overall and progression-free survival [25–29], few have thoroughly evaluated their effects on the spectrum of symptom experiences or irAEs experienced by people on ICIs. This line of inquiry is important given the complex interplay between irAEs and ICI efficacy [40] whereby emerging evidence suggests irAEs may paradoxically correlate with improved clinical outcomes [41]. Although the underpinning mechanisms for this relationship remain unclear, it underscores the need to prioritise management of irAEs and maintenance of QoL concurrently. Current guidelines recommend corticosteroids for irAEs grade 2 and above, yet these treatments introduce secondary complications (e.g., bone loss, weight gain, mood disturbances) that further compromise QoL and treatment adherence [10]. Supportive care modalities, including structured exercise programs and dietary interventions, may mitigate corticosteroid-related toxicities and enhance patients’ capacity to tolerate both irAEs and their treatments. However, sustainable strategies to balance irAE management with treatment efficacy remain underexplored.

### Exercise

Owing to the culmination of > 15 years of research, exercise has been shown to enhance outcomes for people with cancer, including reducing incidence, recurrence, morbidity, and mortality and improving QoL and treatment-related side effects [42, 43]. Observational and pre-clinical research suggests PA and exercise may improve immunotherapy outcomes. For example, Liu et al. [32] found that active (*n* = 31) patients with unresectable hepatocellular carcinoma undergoing combined lenvatinib plus anti-PD-1 therapy had significantly longer overall- (82.7% vs. 67.1%, *p* < 0.05) and progression-free survival (82% vs. 23%, *p* < 0.001) than patients in the sedentary group. Mechanisms underpinning this response are thought to be the ability of exercise to overcome common barriers to immunotherapy efficacy (e.g., dysfunctional immune systems, immunosuppressive tumour microenvironment, and abnormal vasculature) thus improving treatment efficacy [32, 44, 45].

To date, exercise has been shown to improve responsiveness through immune mediated pathways in pancreatic [46], breast [47], non-small cell lung cancer [48], and liver [32] cancer models. Recently, Kurz et al. [46] demonstrated the ability of exercise to sensitise a pancreatic tumour model to αPD-1 immunotherapy observing improved anti-tumour and survival benefits. In this model, exercise was responsible for the promotion of immune cell mobilisation and an increase in intratumoural CD8+ T cells which was mediated by IL-15 release. In humans, acute bouts of exercise mainly high intensity, has elicited significant increases in systemic IL-15 and other myokines (e.g., IL-6, oncostatin-m) that have been implicated in the suppression of cancer growth in pre-clinical and in-vitro models [49–51]. Observational evidence highlights the potential importance of exercise intensity in influencing outcomes for patients undergoing ICI [31]. Verheijden et al. [31] observed a significant association between moderate and vigorous PA and severe irAE incidence (*p* = 0.012) and survival (*p* = 0.036) but not with light intensity exercise. A recent meta-analysis supports this notion demonstrating that 2.5 h per week of high-intensity exercise reduced cancer mortality by 27% compared to a 13% reduction with moderate-intensity [52]. Collectively, this evidence supports the ability of exercise to enhance immune function and ICI responsiveness and the potential importance of intensity, justifying future clinical trials exploring the effect of moderate-high intensity exercise on clinical outcomes for people undergoing ICIs in humans.

Despite promising pre-clinical and observational evidence supporting the role of exercise in improving ICI efficacy, there is a lack of human RCTs or pilot data that also considers irAEs and immunotherapy side effects [32, 46–48]. This review identified three interventional trials exploring the effect of exercise in people undergoing ICIs [25–27]. All three interventions were safe and mostly feasible and demonstrated preliminary efficacy at improving fatigue [25] physical function [27] overall-, and progression-free survival [26]. All interventions were conducted online; one was entirely self-directed and two employed a hybrid model that included group online sessions, self-directed exercise, and behavior change coaching. Supervised interventions appeared superior meeting *a priori* feasibility criteria and presenting higher recruitment (48%), completion (91%), and attendance rates (88%) compared to the hybrid and unsupervised programs used by Charles et al. and Caballero-Borrego et al. [25–27]. Both Charles et al. [25] and Caballero-Borrego et al. [26]. reported challenges with recruitment, uptake, and adherence to unsupervised exercise. This may be related to the unsupervised nature of their self-directed components and/or the prescription that far exceeds the exercise oncology guidelines by Caballero-Borrego et al. (420 min of walking p.w.) [53].

A finding across all three exercise interventions was that treatment-related side effects and self-direction hindered participant recruitment and program completion, whilst supervision facilitated adherence. Charles et al. and Crosby et al. both reported high adherence to supervised components (79–91%) and the most common reasons for missed sessions were due to treatment-related side effects [27, 54]. This is suggestive of the influence of supervision on participants’ desire to engage with and complete exercise interventions and the importance of symptom monitoring and management to enable exercise participation. Participants also reported high levels of satisfaction and highlighted the enjoyment of the supervised group sessions [25]. These barriers to participation and adherence in exercise interventions align with the wider literature, with a recent systematic review highlighting treatment-related side effects, lack of time, and fatigue to be the most common barriers to exercise across all cancer types [55]. Facilitators included improved physical and psychosocial functioning, social engagement, and supervision, which aligns with the findings from this review.

Pre-clinical and observational evidence support the inclusion of exercise in a supportive care intervention for people undergoing immunotherapy. However, further research is needed to explore the effect of exercise on irAEs and consideration needs to be given to intervention design to enhance participation. These findings support the consideration of supervision and group-based sessions in intervention design and early symptom screening, education, and management to facilitate adherence to exercise interventions.

### Diet

Nutrition interventions have a strong body of evidence supporting their effectiveness in managing cachexia and sarcopenia, within the contexts of chemotherapy, hormone therapy, and radiation therapy [56–58]. Cancer-related malnutrition has significant clinical-impacts that negatively affect treatment completion, toxicity, and reduce QoL and survival [59, 60]. Furthermore, conditions such as sarcopenia have been associated with frequency of severe irAEs (65% vs. 34%, *p* = 0.030), fatigue (*p* = 0.011), and linked to a higher rate of dose limiting toxicities (37.5% vs. 10.4%, *p* = 0.011) in people undergoing ipilimumab treatment [8]. Skeletal muscle mass and nutritional status have also been associated with nivolumab response rate, overall and progression-free survival [61].

Findings of this review suggest that dietary patterns and probiotic supplementation might render therapeutic effects for people undergoing immunotherapy through modulation of the gastrointestinal microbiota [36]. There have been rapid developments over the last decade in the ability of the gut microbiota to influence clinical outcomes and treatment toxicities, particularly in the ICI and stem cell transplant settings [40, 62, 63]. Furthermore, a recent meta-analysis highlighted the ability of probiotics to prolong survival and enhance response-rate in people receiving ICIs (all: *p* < 0.001) [21].

The gut microbiome is highly responsive and has a profound influence on the host’s immune system; therefore, it is unsurprising that the gut microbiota has been linked to ICI treatment response [63] and the incidence and severity of irAEs [64]. A recent meta-analysis reported progression-free and overall survival were negatively impacted by antibiotic use [65], suggesting that the disruption of the gut microbiota’s natural state negatively impacts anti-cancer immunity, subsequently leading to poorer clinical outcomes. Furthermore, gut microbiota produces anti-inflammatory short chain fatty acids that have protective effects on the gastrointestinal tract which may be beneficial in the management of gastrointestinal irAEs [66]. Observational evidence also suggests that characteristics within the gut microbiome may predict ICI responsiveness, irAEs, and survival [37]. Given that the gut comprises a highly malleable ecosystem of microorganisms that may be manipulated by both diet and exercise, it serves as a potential target for supportive care interventions to improve irAEs [40, 67].

Despite the paucity of interventional evidence supporting dietary interventions in ICI settings, there is some observational evidence suggesting the positive relationship between improved ICI clinical outcomes and the Mediterranean diet [34], dietary fibre, omega-3 intake [37], and vitamin D supplementation [35]. In other cancer populations, the Mediterranean diet has improved fatigue, skeletal muscle mass, body fat, systemic inflammation, QoL, breast cancer incidence, and chemotherapy-induced nausea and vomiting [58, 68]. Likewise dietary fibre and vitamin D intake has been associated with improved symptom burden including radiotherapy-induced diarrhea and constipation [69], chemotherapy-induced peripheral neuropathy [70], and reduced colorectal cancer-related fatigue [71].

Clinical and mechanistic studies explaining underlying mechanisms of action support these dietary strategies for ICI outcomes. For example, the Mediterranean diet, dietary fibre, omega-3, and vitamin D are suggested to have antioxidant and anti-inflammatory properties that are beneficial in optimising immune function and reducing the inflammation associated with many treatment-related toxicities, such as fatigue and nausea [72]. Furthermore, an inflammatory diet has been found to increase all-cause mortality by 34% in cancer survivors [72]. Thus, encouraging diets with adequate dietary fibre, omega-3, and vitamin D intakes that incorporate Mediterranean diet concepts remain a priority for the clinical care of people with cancer undergoing ICIs [73]. However, to the best of the author’s knowledge there is limited evidence supporting the feasibility and efficacy of these diets in an immunotherapy setting [74].

The identified potential clinical benefits of dietary interventions and paucity of interventional studies that pilot dietary interventions in immunotherapy populations provides a convincing rationale for future research. Prior to nutrition interventions being recommended in clinical care, future well-powered clinical trials are warranted to determine efficacy. Therefore, future interventional research would benefit from expanding the wide base of observational evidence exploring the relationship between factors such as gut microbiome, fibre, and omega-3 intake and improvement in symptom burden and clinical outcomes.

### Symptom management and multimodal interventions

Only one study explored a multimodal supportive care intervention, which was found to be feasible, acceptable, and efficacious for patients with metastatic melanoma [28]. The high overall adherence to the exercise component (85%) was attributed to the tailored program design and supervision, as found in previous studies [25, 27]. Importantly, this intervention reported a 50% (fatigue), 85% (sleep disturbance), and 100% (general aches/pains) reduction in the frequency of the treatment-related side effects [28]. Current literature demonstrates regular symptom monitoring as beneficial; a recent RCT demonstrated a significant reduction in serious irAEs (HR, 0.30 [95% CI, 0.11–0.85]; *p* = 0.02), better QoL, and a reduced rate of treatment discontinuation in people undergoing ICIs who received weekly symptom monitoring and regular contact with allied health clinicians [75]. Interestingly, this was the only study that included psychological intervention despite psychological distress being a recognized unmet need for this population [76]; however, only two participants elected to participate in this modality. Therefore, multimodal interventions which incorporate comprehensive symptom assessment and management may offer a promising strategy to overcome recruitment and adherence challenges for non-pharmacological interventions, such as exercise and should be explored in future research.

## Future directions and conclusion

Although supportive care is increasingly being recognised as essential in oncology, there remain substantial gaps in research and clinical practice [12]. People undergoing ICIs are currently reporting significant unmet needs which include physical changes such as itchy and dry skin, fatigue, and arthralgias as well as psychological toxicities such as anxiety and fear of recurrence [76]. These various unmet needs have also been associated with poor treatment outcomes [77]. Whilst exercise, dietary, and symptom assessment and management shows promise at improving the QoL, symptom burden, and clinical outcomes for people undoing ICIs, there is a notable lack of RCT level evidence, with current interventions focusing on feasibility. This limits the conclusions that can be made on the benefits of wellness interventions and highlights the need for future research. Furthermore, many of these supportive care interventions are currently not standard of care in Australia, thus consideration for the implementation of these modalities must be considered if successful after RCT evidence.

**Fig. 2:**
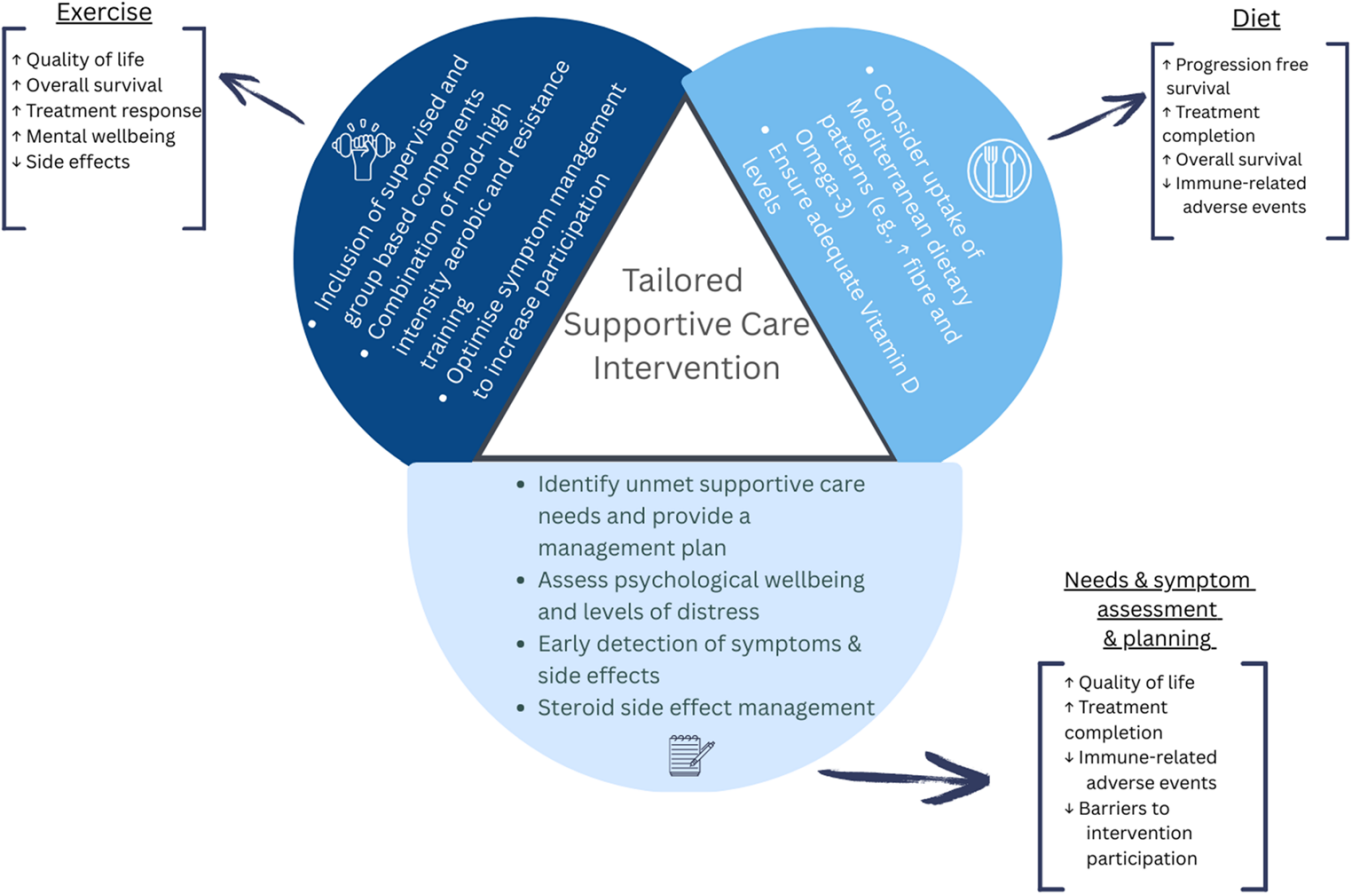
This figure proposes an evidence-based tailored supportive care intervention based upon the findings of this scoping review. The intervention comprises of three key pillars which are supported by mechanistic underpinnings, pre-clinical, observational, and pilot data. *Exercise *will be included to manage immune-checkpoint inhibitor side effects, the side effects of corticosteroid use, and improve clinical outcomes. Based upon the finding of this review the exercise intervention should include supervised components, group-based sessions, and a mix of moderate-high intensity aerobic exercise and resistance training. Dietary advice is reccomended to be included to manage gastrointestinal toxicities and improve clinical outcomes. Dietary advice should be tailored to ensure adequate vitamin and mineral consumption and reccomend diets high in fibre and omega-3 which lends itself to a Mediterranean dietary pattern. *Needs and symptom assessment and planning *is key to reducing immune-related adverse events, improving treatment outcomes, and overcoming barriers to participating in other supportive care modalities. This assessment will include identification of unmet needs and a tailored symptom management plan. *The circles represent key components and boxes potential outcomes. QoL: quality of life, ICI: immune-checkpoint inhibitors, SE’s: side effects, AE: aerobic exercise, RT: resistance training, GIT: gastrointestinal, GMB: gut microbiome, TC: treatment completion, irAEs: immune-related adverse events

There are currently plausible mechanistic underpinnings, pre-clinical, observational, and preliminary pilot evidence to support the further investigation into the feasibility and efficacy of diet and exercise interventions for people undergoing ICIs. Given the relationships discussed in this review between diet, exercise, immune function, and clinical outcomes and the reported barriers to participation and adherence to exercise interventions we suggest a multimodal intervention may address these factors. Based upon the findings of this review future research should aim to explore the feasibility and efficacy of a multimodal intervention that includes symptom assessment and management. Further, it is recommended the design of this intervention takes into consideration the findings of this review including Mediterranean dietary patterns which include high fibre, vitamin D, and omega-3, supervised group-based exercise at a moderate-high intensity, and symptom management/assessment. The proposed intervention can be seen in Fig. 2. If successful pre-clinical and observational evidence suggests this multimodal intervention may improve clinical outcomes such as survival, reduce symptom burden and irAEs, and increase treatment efficacy.

## Key References

- Barron, C.C., et al., *Chronic immune-related adverse events in patients with cancer receiving immune checkpoint inhibitors: a systematic review.* J Immunother Cancer, 2023. **11**(8).
  - ○ A important systematic review that highlights the clinical successes of immune-checkpoint inhibitors and charaterises the rates of immune-related adverse events, and the duration people experienced these for. Lastly, it provided a summary of the treatment required e.g., treatment discontinuation or steriod use. This is the seminal systematic review to summarise this data and highlights the need for management strategies.
- Fang, Q., et al., *Predictors of severity and onset timing of immune-related adverse events in cancer patients receiving immune checkpoint inhibitors: a retrospective analysis.* Front Immunol, 2025. **16**: p. 1,508,512.
  - ○ This paper (*n* = 3795) highlights the predictors of immune-related adverse events and highlights who may be more susceptible to these.
- Lai-Kwon, J., et al., *Selecting Immune Checkpoint Inhibitor Side Effects for Real-Time Monitoring in Routine Cancer Care: A Modified Delphi Study.* JCO Oncol Pract, 2024: p. Op2400037.
  - ○ This is a highly-important paper as it is a recent delphi that identifies current side affects to immune-checkpoint inhibitors and those that are most impactful to patients, carers, and clinicians. This paper also highlights that current symptom monitoring questionaires do not capture the breadth of side effects experienced by people undergoing immune-checkpoint inhibitors and needs to be improved.
- Scotte, F., A. Taylor, and A. Davies, *Supportive Care: The “Keystone” of Modern Oncology Practice.* Cancers (Basel), 2023. **15**(15).
  - ○ This paper is highly important as it provides an overview of supportive care per the definition by The Multinational Association of Supportive Care in Cancer and what components make up a supportive care intervention.
- Hart, N.H., et al., *Survivorship Care for People Affected by Advanced or Metastatic Cancer: MASCC-ASCO Standards and Practice Recommendations.* JCO Oncol Pract, 2024. **20**(9): p. 1160–1172.
  - ○ Given many people undergoing immunotherapy have an advanced/metastatic cancer this paper is important as it highlights the role of survivorship care for this population.
- Liu, X.F., et al., *Physical activity improves outcomes of combined lenvatinib plus anti-PD-1 therapy in unresectable hepatocellular carcinoma: a retrospective study and mouse model.* Exp Hematol Oncol, 2022. **11**(1): p. 20.
  - ○ This paper highlights the ability of exercise to improve treatment response to immune checkpoint inhibitors. The authors first observed associations between physical activity levels and overall survival, progression free survival, and overall response rate. They then looked to confirm this association in a pre-clinical experimental model and observed signficantly suppressed tumour growth and prolonged survival which was mediated by increases in tumour infiltrating lymphocytes provding rationale for the inclusion of exercise in a model of care.
- Wardill, H.R., et al., *Dual contribution of the gut microbiome to immunotherapy efficacy and toxicity: supportive care implications and recommendations.* Support Care Cancer, 2022. **30**(8): p. 6369–6373.
  - ○ This paper highlights the role of the gut microbiome and diet in modulating the response to immune-checkpoint inhibitors and provides rational for including dietary interventions that can influence the gut microbiome.
- Bolte, L.A., et al., *Association of a Mediterranean Diet With Outcomes for Patients Treated With Immune Checkpoint Blockade for Advanced Melanoma.* JAMA Oncol, 2023. **9**(5): p. 705–709.
  - ○ This is an important study as it highlights which dietary patterns are associated with improved clinical outcomes during immune checkpoint inhibitors.
- Crosby, B.J., et al., *Feasibility of supervised telehealth exercise for patients with advanced melanoma receiving checkpoint inhibitor therapy.* Cancer Med, 2023. **12**(13): p. 14,694–14,706.
  - ○ Preliminary pilot evidence supporting the feasibility, efficacy, and safety of a telehealth exercise intervention for people undergoing immune-checkpoint inhibitors. Demonstrates the superior benefits of supervised model and reasons for missed sessions that can be used to guide the development of future interventions.
- Charles, C., et al., *Delivering adapted physical activity by videoconference to patients with fatigue under immune checkpoint inhibitors: Lessons learned from the PACTIMe-FEAS feasibility study.* J Telemed Telecare, 2023. **29**(9): p. 716–724.
  - ○ Preliminary pilot evidence supporting the efficacy and safety of a mixed supervised and unsupervised telehealth exercise intervention for people undergoing immune-checkpoint inhibitors. This intervention include both sueprvised and unsupervised compoentns. The authors identified that the unsupervised components were poorly adhered too and the most common reason was symptoms. This highlights the need for symptom management to assist in overcoming barriers to adhering to exercise interventions.

## Supplementary Information

Below is the link to the electronic supplementary material.

Supplementary Material 1

Supplementary Material 2

## Data Availability

Data is provided within the manuscript or supplementary information files.
