# Supplementary material for: Non-pharmacological Supportive Care Interventions during Immunotherapy for People with Cancer: A Systematic Scoping Review and Future Directions
Source: Curr Oncol Rep. 2025 Dec 6;27(12):1507–23. doi: 10.1007/s11912-025-01714-x (PMC12743693; doi:10.1007/s11912-025-01714-x)
Supplement: Supplementary file 1 — Supplementary Material 1 [file 11912_2025_1714_MOESM1_ESM.docx]

# Supplementary Material 2 – Search Strategy

**Database Searches**

**Date searched** – 25 October 2024

**PubMed** 1412 results

Includes MeSH

**(**"Immunotherapy"[Mesh] OR "Immune Checkpoint Inhibitors"[Mesh] OR "Antibodies, Monoclonal"[Mesh] OR "Antineoplastic Agents, Immunological"[Mesh] OR “immunotherapy”[tiab] OR “immunotherapies”[tiab] OR “checkpoint inhibitor”[tiab] OR “checkpoint inhibitors”[tiab] OR “checkpoint blocker”[tiab] OR “checkpoint blockers”[tiab] OR “monoclonal antibody”[tiab] OR “monoclonal antibodies”[tiab] OR “immunotherapeutic”[tiab] OR “pembrolizumab”[tiab] OR “daratumumab”[tiab] OR “obinutuxumab”[tiab] OR “obinutuzumab”[tiab] OR “nivolumab”[tiab] OR “anti-PD1”[tiab] OR “anti PD1”[tiab] OR “anti-PD-1”[tiab] OR “anti PD-1”[tiab] OR “anti-PD 1”[tiab] OR “anti PD 1”[tiab] OR “anti-CTLA”[tiab] OR “anti CTLA”[tiab]**) AND (**“resistance training”[ti] OR “aerobic training”[ti] OR “interval training”[ti] OR “strength training”[ti] OR “wellness”[ti] OR “wellbeing”[ti] OR “well-being”[ti] OR “well being”[ti] OR “diet”[ti] OR “diets”[ti] OR “nutrition”[ti] OR “dietary”[ti] OR “nutritional”[ti] OR “probiotic”[ti] OR “probiotics”[ti] OR “pro-biotic”[ti] OR “pro biotic”[ti] OR “pro-biotics”[ti] OR “pro biotics”[ti] OR “prebiotic”[ti] OR “prebiotics”[ti] OR “pre-biotic”[ti] OR “pre biotic”[ti] OR “pre-biotics”[ti] OR “pre biotics”[ti] OR “non-pharmacological”[ti] OR “non pharmacological”[ti] OR “nonpharmacological”[ti] OR “complementary”[ti] OR “exercise”[ti] OR “walking”[ti] OR “physical activity”[ti] OR “lifestyle”[ti] OR “supportive care”[ti] OR “supplement”[ti] OR “supplements”[ti] OR “supplementation”[ti] OR “psychology”[ti] OR “psychological”[ti] OR “meditation”[ti] OR “mindfulness”[ti] OR “breathwork”[ti] OR “breath work”[ti] OR “acupuncture”[ti] OR “yoga”[ti] OR “holistic”[ti]**) AND (**eng[la] OR und[la]**) AND** 2014:2025[dp] **NOT (**animals [mh] NOT humans [mh]**)**

**CINAHL Complete (EBSCOhost)** 461 results

Includes CINAHL Subject Headings

**(**MH "Immunotherapy+" OR MH "Immune Checkpoint Inhibitors+" OR MH "Antibodies, Monoclonal" OR TI(“immunotherapy” OR “immunotherapies” OR “checkpoint inhibitor” OR “checkpoint inhibitors” OR “checkpoint blocker” OR “checkpoint blockers” OR “monoclonal antibody” OR “monoclonal antibodies” OR “immunotherapeutic” OR “pembrolizumab” OR “daratumumab” OR “obinutuxumab” OR “obinutuzumab” OR “nivolumab” OR “anti-PD1” OR “anti PD1” OR “anti-PD-1” OR “anti PD-1” OR “anti-PD 1” OR “anti PD 1” OR “anti-CTLA” OR “anti CTLA”) OR AB(“immunotherapy” OR “immunotherapies” OR “checkpoint inhibitor” OR “checkpoint inhibitors” OR “checkpoint blocker” OR “checkpoint blockers” OR “monoclonal antibody” OR “monoclonal antibodies” OR “immunotherapeutic” OR “pembrolizumab” OR “daratumumab” OR “obinutuxumab” OR “obinutuzumab” OR “nivolumab” OR “anti-PD1” OR “anti PD1” OR “anti-PD-1” OR “anti PD-1” OR “anti-PD 1” OR “anti PD 1” OR “anti-CTLA” OR “anti CTLA”)**) AND (**TI(“resistance training” OR “aerobic training” OR “interval training” OR “strength training” OR “wellness” OR “wellbeing” OR “well-being” OR “well being” OR “diet” OR “diets” OR “nutrition” OR “dietary” OR “nutritional” OR “probiotic” OR “probiotics” OR “pro-biotic” OR “pro biotic” OR “pro-biotics” OR “pro biotics” OR “prebiotic” OR “prebiotics” OR “pre-biotic” OR “pre biotic” OR “pre-biotics” OR “pre biotics” OR “non-pharmacological” OR “non pharmacological” OR “nonpharmacological” OR “complementary” OR “exercise” OR “walking” OR “physical activity” OR “lifestyle” OR “supportive care” OR “supplement” OR “supplements” OR “supplementation” OR “psychology” OR “psychological” OR “meditation” OR “mindfulness” OR “breathwork” OR “breath work” OR “acupuncture” OR “yoga” OR “holistic”)**) AND (**LA English**) AND** PY 2014-2025 **NOT (**(MH "Animals+" OR MH "Animal Studies" OR TI animal model*) NOT MH "Human"**)**

**APA PsycInfo (EBSCOhost)** 21 results

Includes APA Thesaurus of Psychological Index Terms

**(**DE "Immunotherapy" OR DE "Monoclonal Antibodies" OR TI(“immunotherapy” OR “immunotherapies” OR “checkpoint inhibitor” OR “checkpoint inhibitors” OR “checkpoint blocker” OR “checkpoint blockers” OR “monoclonal antibody” OR “monoclonal antibodies” OR “immunotherapeutic” OR “pembrolizumab” OR “daratumumab” OR “obinutuxumab” OR “obinutuzumab” OR “nivolumab” OR “anti-PD1” OR “anti PD1” OR “anti-PD-1” OR “anti PD-1” OR “anti-PD 1” OR “anti PD 1” OR “anti-CTLA” OR “anti CTLA”) OR AB(“immunotherapy” OR “immunotherapies” OR “checkpoint inhibitor” OR “checkpoint inhibitors” OR “checkpoint blocker” OR “checkpoint blockers” OR “monoclonal antibody” OR “monoclonal antibodies” OR “immunotherapeutic” OR “pembrolizumab” OR “daratumumab” OR “obinutuxumab” OR “obinutuzumab” OR “nivolumab” OR “anti-PD1” OR “anti PD1” OR “anti-PD-1” OR “anti PD-1” OR “anti-PD 1” OR “anti PD 1” OR “anti-CTLA” OR “anti CTLA”)**) AND (**TI(“resistance training” OR “aerobic training” OR “interval training” OR “strength training” OR “wellness” OR “wellbeing” OR “well-being” OR “well being” OR “diet” OR “diets” OR “nutrition” OR “dietary” OR “nutritional” OR “probiotic” OR “probiotics” OR “pro-biotic” OR “pro biotic” OR “pro-biotics” OR “pro biotics” OR “prebiotic” OR “prebiotics” OR “pre-biotic” OR “pre biotic” OR “pre-biotics” OR “pre biotics” OR “non-pharmacological” OR “non pharmacological” OR “nonpharmacological” OR “complementary” OR “exercise” OR “walking” OR “physical activity” OR “lifestyle” OR “supportive care” OR “supplement” OR “supplements” OR “supplementation” OR “psychology” OR “psychological” OR “meditation” OR “mindfulness” OR “breathwork” OR “breath work” OR “acupuncture” OR “yoga” OR “holistic”)**) AND (**LA English**) AND** PY 2014-2025

**Embase (Elsevier)** 1657 results

Includes Emtree

**(**'immunotherapy'/exp/mj OR 'immune checkpoint inhibitor'/exp/mj OR 'monoclonal antibody'/exp/mj OR 'immunological antineoplastic agent'/exp/mj OR “immunotherapy”:ti,ab OR “immunotherapies”:ti,ab OR “checkpoint inhibitor”:ti,ab OR “checkpoint inhibitors”:ti,ab OR “checkpoint blocker”:ti,ab OR “checkpoint blockers”:ti,ab OR “monoclonal antibody”:ti,ab OR “monoclonal antibodies”:ti,ab OR “immunotherapeutic”:ti,ab OR “pembrolizumab”:ti,ab OR “daratumumab”:ti,ab OR “obinutuxumab”:ti,ab OR “obinutuzumab”:ti,ab OR “nivolumab”:ti,ab OR “anti-PD1”:ti,ab OR “anti PD1”:ti,ab OR “anti-PD-1”:ti,ab OR “anti PD-1”:ti,ab OR “anti-PD 1”:ti,ab OR “anti PD 1”:ti,ab OR “anti-CTLA”:ti,ab OR “anti CTLA”:ti,ab**)** **AND (**“resistance training”:ti OR “aerobic training”:ti OR “interval training”:ti OR “strength training”:ti OR “wellness”:ti OR “wellbeing”:ti OR “well-being”:ti OR “well being”:ti OR “diet”:ti OR “diets”:ti OR “nutrition”:ti OR “dietary”:ti OR “nutritional”:ti OR “probiotic”:ti OR “probiotics”:ti OR “pro-biotic”:ti OR “pro biotic”:ti OR “pro-biotics”:ti OR “pro biotics”:ti OR “prebiotic”:ti OR “prebiotics”:ti OR “pre-biotic”:ti OR “pre biotic”:ti OR “pre-biotics”:ti OR “pre biotics”:ti OR “non-pharmacological”:ti OR “non pharmacological”:ti OR “nonpharmacological”:ti OR “complementary”:ti OR “exercise”:ti OR “walking”:ti OR “physical activity”:ti OR “lifestyle”:ti OR “supportive care”:ti OR “supplement”:ti OR “supplements”:ti OR “supplementation”:ti OR “psychology”:ti OR “psychological”:ti OR “meditation”:ti OR “mindfulness”:ti OR “breathwork”:ti OR “breath work”:ti OR “acupuncture”:ti OR “yoga”:ti OR “holistic”:ti**) AND** [english]/lim **AND** [2014-2025]/py **NOT (**‘animal experiment’/de NOT (‘human experiment’/de OR ‘human’/de)**)**

**Cochrane Library (Wiley) – CENTRAL** (408 Trials)

Includes MeSH

*Advanced Search > Search manager*

Limited to *CENTRAL Trials only Original publication year* from 2014 to present

ID Search Hits

#1 MeSH descriptor: [Immunotherapy] explode all trees 12295

#2 MeSH descriptor: [Immune Checkpoint Inhibitors] explode all trees 295

#3 MeSH descriptor: [Antibodies, Monoclonal] explode all trees 22193

#4 MeSH descriptor: [Antineoplastic Agents, Immunological] explode all trees 1147

#5 (“immunotherapy” OR “immunotherapies” OR “checkpoint inhibitor” OR “checkpoint inhibitors” OR “checkpoint blocker” OR “checkpoint blockers” OR “monoclonal antibody” OR “monoclonal antibodies” OR “immunotherapeutic” OR “pembrolizumab” OR “daratumumab” OR “obinutuxumab” OR “obinutuzumab” OR “nivolumab” OR “anti PD1” OR “anti PD 1” OR “anti CTLA”):ti,ab 31729

#6 #1 OR #2 OR #3 OR #4 OR #5 56941

#7 (“resistance training” OR “aerobic training” OR “interval training” OR “strength training” OR “wellness” OR “wellbeing” OR “well being” OR “diet” OR “diets” OR “nutrition” OR “dietary” OR “nutritional” OR “probiotic” OR “probiotics” OR “pro biotic” OR “pro biotics” OR “prebiotic” OR “prebiotics” OR “pre biotic” OR “pre biotics” OR “non pharmacological” OR “nonpharmacological” OR “complementary” OR “exercise” OR “walking” OR “physical activity” OR “lifestyle” OR “supportive care” OR “supplement” OR “supplements” OR “supplementation” OR “psychology” OR “psychological” OR “meditation” OR “mindfulness” OR “breathwork” OR “breath work” OR “acupuncture” OR “yoga” OR “holistic”):ti 188670

#8 #6 AND #7 with Publication Year from 2014 to present, in Trials 408

**Web of Science (Clarivate)** 951 results

Web of Science Core Collection (1900-present), All Editions

*Advanced search* (entered directly in *Query Preview* box)

Refined results to Publication years from 2014 to 2025 and English language


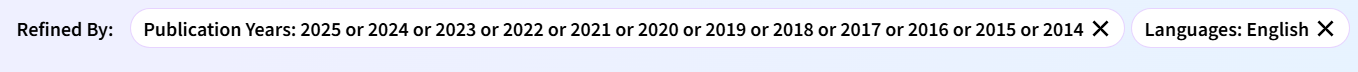


**(**TI=(“immunotherapy” OR “immunotherapies” OR “checkpoint inhibitor” OR “checkpoint inhibitors” OR “checkpoint blocker” OR “checkpoint blockers” OR “monoclonal antibody” OR “monoclonal antibodies” OR “immunotherapeutic” OR “pembrolizumab” OR “daratumumab” OR “obinutuxumab” OR “obinutuzumab” OR “nivolumab” OR “anti-PD1” OR “anti PD1” OR “anti-PD-1” OR “anti PD-1” OR “anti-PD 1” OR “anti PD 1” OR “anti-CTLA” OR “anti CTLA”) OR AB=(“immunotherapy” OR “immunotherapies” OR “checkpoint inhibitor” OR “checkpoint inhibitors” OR “checkpoint blocker” OR “checkpoint blockers” OR “monoclonal antibody” OR “monoclonal antibodies” OR “immunotherapeutic” OR “pembrolizumab” OR “daratumumab” OR “obinutuxumab” OR “obinutuzumab” OR “nivolumab” OR “anti-PD1” OR “anti PD1” OR “anti-PD-1” OR “anti PD-1” OR “anti-PD 1” OR “anti PD 1” OR “anti-CTLA” OR “anti CTLA”)**)** **AND (**TI=(“resistance training” OR “aerobic training” OR “interval training” OR “strength training” OR “wellness” OR “wellbeing” OR “well-being” OR “well being” OR “diet” OR “diets” OR “nutrition” OR “dietary” OR “nutritional” OR “probiotic” OR “probiotics” OR “pro-biotic” OR “pro biotic” OR “pro-biotics” OR “pro biotics” OR “prebiotic” OR “prebiotics” OR “pre-biotic” OR “pre biotic” OR “pre-biotics” OR “pre biotics” OR “non-pharmacological” OR “non pharmacological” OR “nonpharmacological” OR “complementary” OR “exercise” OR “walking” OR “physical activity” OR “lifestyle” OR “supportive care” OR “supplement” OR “supplements” OR “supplementation” OR “psychology” OR “psychological” OR “meditation” OR “mindfulness” OR “breathwork” OR “breath work” OR “acupuncture” OR “yoga” OR “holistic”)**)**

**Scopus (Elsevier)** 877 results

Advanced search

**(**TITLE-ABS(“immunotherapy” OR “immunotherapies” OR “checkpoint inhibitor” OR “checkpoint inhibitors” OR “checkpoint blocker” OR “checkpoint blockers” OR “monoclonal antibody” OR “monoclonal antibodies” OR “immunotherapeutic” OR “pembrolizumab” OR “daratumumab” OR “obinutuxumab” OR “obinutuzumab” OR “nivolumab” OR “anti-PD1” OR “anti PD1” OR “anti-PD-1” OR “anti PD-1” OR “anti-PD 1” OR “anti PD 1” OR “anti-CTLA” OR “anti CTLA”)**)** AND **(**TITLE(“resistance training” OR “aerobic training” OR “interval training” OR “strength training” OR “wellness” OR “wellbeing” OR “well-being” OR “well being” OR “diet” OR “diets” OR “nutrition” OR “dietary” OR “nutritional” OR “probiotic” OR “probiotics” OR “pro-biotic” OR “pro biotic” OR “pro-biotics” OR “pro biotics” OR “prebiotic” OR “prebiotics” OR “pre-biotic” OR “pre biotic” OR “pre-biotics” OR “pre biotics” OR “non-pharmacological” OR “non pharmacological” OR “nonpharmacological” OR “complementary” OR “exercise” OR “walking” OR “physical activity” OR “lifestyle” OR “supportive care” OR “supplement” OR “supplements” OR “supplementation” OR “psychology” OR “psychological” OR “meditation” OR “mindfulness” OR “breathwork” OR “breath work” OR “acupuncture” OR “yoga” OR “holistic”)**)** **AND** PUBYEAR AFT 2013 **AND** Language(English)
